# A retroperitoneal mass presenting as acute scrotum: a rare presentation and review of the literature

**DOI:** 10.1093/jscr/rjaa575

**Published:** 2021-01-18

**Authors:** Ali Alyami, Abdullah Alkhayal

**Affiliations:** College of Medicine, King Saud bin Abdulaziz University for Health Sciences, Riyadh, Saudi Arabia; College of Medicine, King Saud bin Abdulaziz University for Health Sciences, Riyadh, Saudi Arabia; Division of Urology, Department of Surgery, Department of Urology, King Abdulaziz Medical City, Riyadh, Saudi Arabia

## Abstract

The acute scrotum is defined as a new-onset pain of the intrascrotal contents. The differential diagnosis of acute scrotum includes a variety of etiologies. We report a case of an 18 years old presented with acute scrotal pain with scrotal ultrasound suggestive of testicular torsion, underwent bilateral orchiopexy for suspected testicular torsion. The patient came later with persistent testicular pain. A computerized tomography (CT) scan of the abdomen and pelvis was done and showed a retroperitoneal mass. We suggest that patients with atypical presentation of the acute scrotum should undergo CT scan of the abdomen and pelvis to rule out retroperitoneal pathologies and referred pain.

## INTRODUCTION

Acute scrotal pain is defined as a sudden onset of pain, swelling, with or without tenderness of the intrascrotal contents. Patients may describe the onset of symptoms occurring within minutes up to 1–2 days, dependent on the etiology [1, 2]. The differential diagnosis of the acute scrotum includes a wide variety of diseases. Rapid evaluation and diagnostics are necessary due to the time dependency of certain morbid conditions such as testicular torsion and strangulated hernia that will require emergency surgical intervention [1]. Other causes of the acute scrotum are torsion of the testicular appendix, epididymitis, epididymo-orchitis, scrotal abscess and testicular cancer [1]. However, non-scrotal conditions are rarely present clinically only as acute scrotum such as renal colic, ruptured aneurysm or other causes of retroperitoneal pathologies [2]. Evaluating acute scrotum sometimes could be a diagnostic challenge and must be carefully evaluated with full patient history, physical examination, laboratory tests and imaging studies [1, 2]. Doppler ultrasonography is the most appropriate imaging modality for acute scrotal evaluation, which has a sensitivity range between 96 and 100% and specificity between 84 and 95% when it does not delay definitive surgical consultation in cases of presumed torsion [1]. In cases of non-scrotal causes of acute scrotum, ultrasound extended to the abdomen, groin and thighs, and further imaging modalities are considered when needed [1, 2].

**Figure 1:**
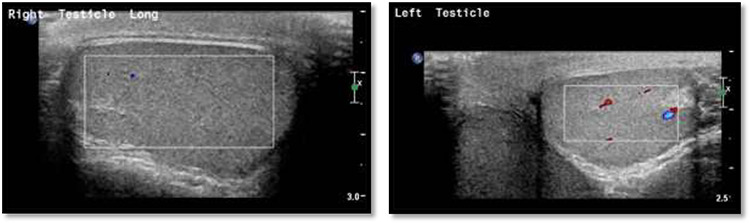
Scrotal Doppler ultrasound shows the size, echogenicity and vascularity is maintained for both testicle with slightly reduced in the right testicle that could be suggestive of right torsion/detorsion.

**Figure 2:**
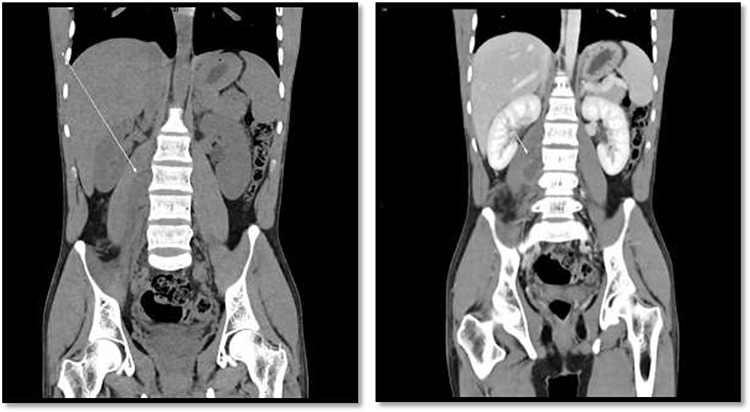
CT scan showed there is 4 × 3 cm lobulated mass noted at the right paravertebral space invading the right psoas muscle at the level of L3 and L4 and extending through the neural foramina, the associated with epidural component compressing the spinal cord from the level of the L2–L4.

## CASE REPORT

**Figure 3:**
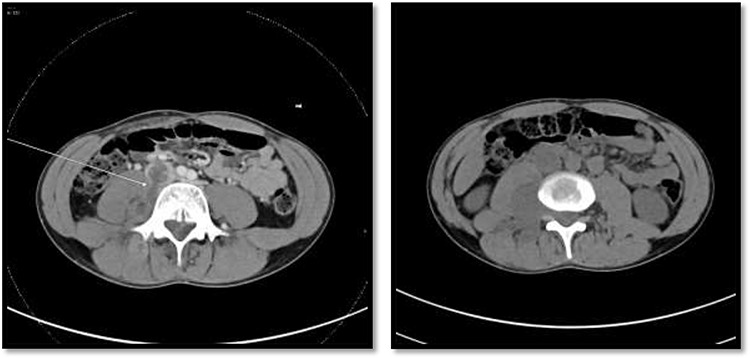
On the left image, there is associated fistulas tract between the mass and infrarenal inferior vena cava with a small hyperdense component may represent hemorrhage or solid component. The right image CT scan showed the mass extending through the neural foramina compressing the spinal cord from the level of the L2–L4.

An 18-year-old healthy man presented to the emergency department (ED) with 1-day history of right testicular pain and vomiting with no fever nor abdominal pain. Physical examination showed mild tenderness in the right testis, no swelling, normal longitudinal position and cremasteric reflex. Scrotal Doppler ultrasound was performed and showed reduced vascularity in the right testis and the left testis was normal. Moreover, the size and echogenicity were maintained for both testes (Fig. 1). Two months prior to this presentation, the patient presented with the same attack with only mild tenderness in the right testis, and all investigations were within normal limits and he was discharged with oral analgesic drugs. The decision was made to undergo surgical exploration for possible torsion/detorsion of the right testis. The patient was transferred immediately to the operating room for bilateral orchiopexy. The right spermatic cord was engorged with no evidence of ischemia identified and bilateral orchiopexy was done successfully. After the surgery, the patient was kept for observation for 24 h and then discharged. After 2 days from the surgery, the patient came back to ED with testicular pain and post-surgical swelling. We decided to do computerized tomography (CT) scan of the abdominal and pelvis to rule out retroperitoneal pathologies or referred pain. The CT showed 4 × 3 cm lobulated mass noted at right paravertebral space invading the right psoas muscle at the level of L3 and L4 (Fig. 2). The CT scan also showed there is an associated fistula tract between the mass and infrarenal inferior vena cava with thrombosis associated with epidural component extending through the neural foramina compressing the spinal cord from the level of the L2–L4 (Fig. 3). The patient was referred to spinal surgery and CT-guided paraspinal lumbar biopsy was taken. The histopathology came with the diagnosis of Ewing sarcoma. The decision was made by the medical oncology to start on a systemic chemotherapy (vincristine + Adriamycin + cyclophosphamide alternating with ifosfamide + etoposide (VAC/IE)) regimen. After six cycles, the follow-up magnetic resonance imaging demonstrated significant resolution of the right psoas and posterior paraspinal metastatic disease. In addition, further resolution with residual intraspinal extradural metastatic disease noted at right L2–3 and L3–4 levels. The patient is still followed up as an outpatient clinic with medical oncology.

## DISCUSSION

The acute scrotum is characterized by intense acute scrotal pain that might be associated with other signs and symptoms such as abdominal pain, inflammation and fever [1, 2]. Accurate and early diagnosis of the acute scrotum is of the utmost importance to avoid testicular loss and/or needless surgery [1]. The differential diagnosis of an acute scrotum includes a wide variety of etiologies that could be related to scrotal conditions such as torsion of the testis or its appendages, epididymo-orchitis or could be related to non-scrotal conditions like retroperitoneal pathologies, renal colic, aneurysm rupture, primary abdominal or pelvic tumors and metastases [1, 2]. The incidence of the acute scrotum as a presenting complaint is not well reported, but male genitourinary complaints are estimated at between 0.5 and 2.5% of all ED visits [1]. A study published by Kim *et al*. [3] reported appendix torsion is a predominantly frequent cause of acute scrotum in childhood and adolescence who visited primary care urology clinics. On the other hand, over 600 000 cases are diagnosed in the US EDs as epididymitis in men between 18 and 50 years of age [4]. Non-scrotal conditions, however, can rarely present clinically only as of the acute scrotum; therefore, reaching the accurate diagnosis could be challenging, which needs more investigations and imaging modalities. The scrotal pain secondary to retroperitoneal mass is probably because of compression, stretching, dislocation, and infiltration of the neuronal ganglia and both the autonomic and somatic routes innervating the testes [2]. The retroperitoneal tumors become clinically manifested when there are large or involve adjacent structures. In the literatures, there are limited cases reported as the same finding as our case. Malde [5] reported a case of a 47-year-old man initially present to the ED with a history of sudden-onset, intermittent left testicular pain with no evidence of systemic symptoms nor family history, and testicular ultrasound scan also failed to show any abnormality in either testis. Their patient was treated with a course of antibiotics for suspected epididymo-orchitis, but this failed to relieve his symptoms. Later, they subsequently thought that he may have a distal ureteric calculus causing referred pain and they underwent for CT scan of his renal tract, which revealed a 10 × 12 cm retroperitoneal mass [5]. Determining the cause of acute scrotum could be sometimes challenging because a wide variety of scrotal and non-scrotal etiologies and extended imaging modalities are needed.

## CONCLUSION

The differential diagnosis of acute scrotum remains difficult in some patients. Non-scrotal conditions such as retroperitoneal mass may present clinically with acute scrotal pain; thus, further extended imaging modalities are considered in patients with atypical presentation.

## CONFLICT OF INTEREST STATEMENT

None declared.

## FUNDING

None.

## CONSENT FOR PUBLICATION

Informed consent was obtained from participant.
